# Prevalence of Pathogenic Germline *BRCA1/2* Variants and Their Association with Clinical Characteristics in Patients with Epithelial Ovarian Cancer in a Rural Area of Japan

**DOI:** 10.3390/genes13061085

**Published:** 2022-06-18

**Authors:** Akiko Abe, Issei Imoto, Shoichiro Tange, Masato Nishimura, Takeshi Iwasa

**Affiliations:** 1 Department of Gynecology, Cancer Institute Hospital of JCFR, Tokyo 135-8550, Japan; 2 Department of Obstetrics and Gynecology, Tokushima University, Tokushima 770-8503, Japan; nishimura.masato@tokushima-u.ac.jp (M.N.); iwasa.takeshi@tokushima-u.ac.jp (T.I.); 3 Aichi Cancer Center Research Institute, Nagoya 464-8681, Japan; issehgen@gmail.com; 4 Department of Medical Genome Sciences, Research Institute for Frontier Medicine, Sapporo Medical University, Sapporo 060-8556, Japan; stange@sapmed.ac.jp

**Keywords:** *BRCA1/2*, epithelial ovarian cancer, germline pathogenic variants, hereditary breast and ovarian cancer syndrome (HBOC)

## Abstract

The prevalence of germline *BRCA1* or *BRCA2* pathogenic variants (g*BRCA1/2*-PV) in patients with primary epithelial ovarian cancer (OC) in a rural area of Japan and their association with clinical characteristics, including treatment response and survival outcome, were investigated. A total of 123 unbiased patients with OC were tested for g*BRCA1* and g*BRCA2* using next-generation sequencing-based targeted amplicon sequencing. Clinical characteristics of OC patients with and without g*BRCA1/2* status were compared. The overall prevalence of g*BRCA1/2*-PV was 15.4% (19 cases), with g*BRCA2*-PV (10.5%, 13 cases) being more common than g*BRCA1*-PV (4.9%, 6 cases). Among the observed g*BRCA1/2*-PV, several novel variants were included, suggesting that g*BRCA1/2*-PV unique to the local area exist. g*BRCA1/2*-PV was significantly more prevalent in OC patients at an older age, with high-grade serous carcinoma, with advanced-stage tumors, and with a family history of breast cancer or hereditary breast and ovarian cancer syndrome (HBOC)-associated cancers. Patients with advanced-stage OC with g*BRCA1/2*-PV showed a significantly lower recurrence rate and tended to have better progression-free and overall survival than those with wild-type g*BRCA1/2*. Genetic testing for g*BRCA1/2* status in all OC patients is useful not only for diagnosing HBOC in patients and their relatives to assess the risk of HBOC-associated cancers, but also to estimate therapy response and outcomes in patients.

## 1. Introduction

Primary ovarian, fallopian tube, and peritoneal carcinoma (OC) remains the most lethal gynecological malignancy among women [1] owing to late-stage diagnosis in most cases, as well as frequent recurrence and treatment resistance [1,2]. Although most cases of OC are sporadic, at least 10% of patients with OC seem to be hereditary and associated with a genetic predisposition, regardless of ethnic background [3,4,5,6,7].

Pathogenic variants (PV) of germline *BRCA1* or *BRCA2* (g*BRCA1/2*), which are hereditary breast and ovarian cancer syndrome (HBOC) susceptibility genes with autosomal dominant inheritance, are the major cause of hereditary OC. The lifetime risk of developing OC by 80 years of age in women with g*BRCA1* and g*BRCA2* PV is approximately 44% and 17%, respectively [8]. Owing to the high penetrance of breast cancer (BC) and OC in women with g*BRCA1/2*-PV, genetic testing for these genes and disclosure of their PV to patients and their relatives through genetic counseling can be useful for identifying women with an OC predisposition for effective prevention strategies and early diagnosis.

The prevalence of g*BRCA1/2*-PV in Japanese patients with OC has been reported in retrospective studies from a single institute in an urban area (27/230 cases, 11.7%) [6] and a multicenter epidemiological survey (93/634 cases, 14.7%) [7]. In Japan, *BRCA1/2* genetic testing for OC was covered by the national health insurance system as a companion diagnostic for Olaparib, a poly(ADP-ribose) polymerase inhibitor, for applicable cases, and as a definitive diagnosis of HBOC for the preventive intervention of secondary cancers in all cases from 2019 and 2020, respectively. Therefore, the number of patients with OC diagnosed with HBOC is expected to rapidly increase in Japan. In South Korea, indeed, *BRCA1/2* genetic testing became supported by National Health Insurance in 2012 for individuals at risk of HBOC, increasing genetic testing recommendations by physicians and the number of *BRCA1/2* genetic tests [9]. However, published data on the prevalence and clinical characteristics of Japanese patients with OC with g*BRCA1/2*-PV remain scarce.

Here, we report a retrospective analysis of the prevalence of g*BRCA1/2*-PV and its impact on clinicopathological characteristics among Japanese patients with OC who underwent next-generation sequencing -based targeted amplicon sequencing (TAS) for the *BRCA1* and *BRCA2* genes in a single institute in a rural area of Japan before the approval of the national health insurance system for g*BRCA1/2* testing to use olaparib in platinum-sensitive recurrent OC or to diagnose HBOC in all patients with OC in Japan. In addition, we aimed to characterize the observed g*BRCA1/2*-PV by comparing with variants previously reported in urban areas [6] and a nationwide multicenter study [7] in Japan.

## 2. Materials and Methods

### 2.1. Population Description

Women with histologically confirmed invasive OC who were treated between 2010 and 2016 at the Tokushima University Hospital located on the island of Shikoku, Japan, were retrospectively recruited for this study. Patients with insufficient clinical and/or pathological data or those who died within less than six months after diagnosis were excluded from the study. In total, 123 patients with OC were enrolled in this study. Self-reported information, including familial cancer history, was collected at study entry. This study was approved by the institutional review board of Tokushima University Hospital, Japan (approval number: 2373), and all participants provided written informed consent for the use of genomic and clinical data for research purposes. When consent was obtained, participants were also asked whether they will be reported for the results of germline gene alteration, respectively, from treating physicians. All procedures were conducted in accordance with the Declaration of Helsinki.

### 2.2. Clinicopathological Data

Demographic and clinicopathological characteristics, such as patients’ menstruation status, personal or familial history of cancer, histologic subtype, and the International Federation of Gynecology and Obstetrics (FIGO) stage, were collected from medical records. Family history was defined as first- and second-degree *BRCA1/2*-associated cancers (breast, ovarian, pancreatic, and/or prostate cancer). The histological diagnosis was performed by two independent pathologists based on the WHO classification 2014 [10]. Treatment information, such as primary treatment strategy (primary debulking surgery [PDS] alone, PDS followed by postoperative adjuvant chemotherapy, or neoadjuvant chemotherapy [NAC] followed by interval debulking surgery [IDS]) and chemotherapy after recurrence, responses to treatments, and prognosis, were also collected from the medical records. The treatment-free interval (TFI) was calculated as the time interval between the date of completion of primary treatment and the date of disease progression. Platinum-sensitive recurrence was defined as a TFI of 6 months or longer, whereas platinum-resistant recurrence was defined as a TFI shorter than 6 months. Follow-up was measured from the date of diagnosis to the date of the last report for live patients. Progression-free survival (PFS) was calculated as the time interval between the date of initial diagnosis and the date of progression (recurrence) or end of follow-up. Overall survival (OS) was calculated as the time interval between the date of initial diagnosis and the date of cancer-related death or end of follow-up.

### 2.3. gBRCA1/2 Analysis

Germline DNA was isolated from whole blood samples using a Gentra Puregene Blood Kit (Qiagen, Hilden, Germany). DNA concentration was measured using a Qubit^®^ instrument (Thermo Fisher Scientific Inc., Waltham, MA, USA).

For TAS, a customized Generead v2 DNAseq Panel (Qiagen) panel was used for library construction, following the manufacturer’s protocol. The entire coding exon regions of *BRCA1* (NM_007294.4) and *BRCA2* (NM_000059.4) were included in the targeted panel. TAS using next-generation sequencing was performed with 2 × 150 bp paired-end reads on an Illumina MiSeq platform (Illumina, San Diego, CA, USA), following the manufacturer’s protocol.

The sequenced reads were mapped to the human genome reference (hg19). Single nucleotide variants and short insertions/deletions were detected using our pipeline for next-generation sequencing data analysis, as described [11], with a minor modification owing to a software update specifically for the bioinformatics pipeline [12]. To identify pathogenic single nucleotide variants, we excluded sequence variants with higher allele frequencies (>0.01) from the 1000 Genomes Project database (https://www.internationalgenome.org/ (accessed on 1 October 2021)), Genome Aggregation Database (gnomAD, https://gnomad.broadinstitute.org/ (accessed on 1 October 2021)), Human Genetic Variation Database (HGVD, https://www.hgvd.genome.med.kyoto-u.ac.jp/ (accessed on 1 Octber 2021)), and Japanese Multi Omics Reference Panel (jMorp, https://jmorp.megabank.tohoku.ac.jp/202008/ (accessed on 1 October 2021)). Copy-number alteration analysis using TAS data was performed as previously described [12,13]. The pathogenicity of each single nucleotide variant or short insertions/deletion was evaluated according to the American College of Medical Genetics and Genomics and the Association for Molecular Pathology (ACMG_AMP guidelines) [14], as well as the pathogenicity assertions registered in ClinVar (https://www.ncbi.nlm.nih.gov/clinvar/ (accessed on 15 March 2022)). In ‘population data’ of the evidence framework in the ACMG_AMP guidelines, each variant was determined to meet the PM2 category using population databases, particularly using the Japanese databases (ToMMo genome variation database v4.7k and HGVD v2.1). In ‘computational and predictive data’, each variant was determined to meet the PVS1, PS1, PM4, PM5, or PP3 category. In ‘functional data’, each variant was determined to meet the PS3 category. We did not evaluate evidence of ‘segregation data’, ‘de novo data’, ‘allelic data’, ‘other database,’ or ‘other data’ in the evidence framework of the ACMG_AMP guidelines. In addition, variants classified as ‘pathogenic’ or ‘likely pathogenic’ by expert panels in the ClinVar database were considered pathogenic or likely pathogenic, respectively. All single nucleotide variants and short insertions/deletions were confirmed using direct Sanger sequencing.

According to the results of the g*BRCA1/2* analysis, two groups of patients, g*BRCA1/2*-PV and g*BRCA1/2*-WT, were determined. Women with variants of unknown significance in g*BRCA1/2* were classified into the g*BRCA1/2*-WT group.

### 2.4. Statistical Analysis

Clinicopathological variables pertaining to the corresponding patients were analyzed using Pearson’s χ^2^-test or Fisher’s exact test. For survival analysis, Kaplan–Meier survival curves were constructed for the groups based on univariate predictors, and differences between groups were tested using a log-rank test. Univariate survival analysis was performed using the likelihood ratio test of the stratified Cox proportional hazard model. All statistical analyses were two-sided, and *p* values < 0.05 were considered statistically significant. Statistical analyses were performed using the JMP software version 14.0.0 (SAS Institute Japan, Tokyo, Japan) or R version 4.0.3 (R Project for Statistical Computing, Vienna, Austria).

## 3. Results

### 3.1. Description of the Study Population

The clinicopathological characteristics of 123 participants are presented in Table 1. The median (range) age at diagnosis was 54 (28–87) years. Histological subtyping revealed that 47/123 (38.2%) and 28/123 (22.8%) cases were high-grade serous carcinoma (HGSC) and clear cell carcinoma, respectively. As per FIGO stages, 54/123 (43.9%) cases were stages III–IV. The characteristics of participants with OC in a combination of the present study and two previously reported larger studies are presented in Supplementary Table S1 [6,7].

### 3.2. gBRCA1/2 PV Detected in OC Cases

Through our TAS analysis, a total of 12 different g*BRCA1/2*-PV were identified in 19 cases among 123 participants (15.4%, Table 1 and Table 2). The prevalence of g*BRCA2*-PV (10.5%, 13/123) was higher than that of g*BRCA1*-PV (4.9%, 6/123). No patient had both g*BRCA1*-PV and g*BRCA2*-PV. The prevalence of g*BRCA1*-PV and g*BRCA2*-PV within the ovarian cancer cluster region (OCCR) was 66.7% (4/6) and 46.2% (6/13), respectively [15].

Two and four PVs of g*BRCA1* (c.3503_3509del and c.5161C>T) and g*BRCA2* (c.5645C>A, c.6952C>T, c.7758G>A and c.7922dup), respectively, were identified in more than one case, although the individuals included in this study were confirmed to be unrelated to each other up to the third degree of kinship.

In g*BRCA1*-PV, c.3503_3509del is within the OCCR, while c.5161C>T is outside of the OCCR. In g*BRCA2*-PV, two variants (c.5645C>A and c.6952C>T) are within the OCCR, while two variants (c.7758G>A and c.7922dup) are outside of the OCCR.

### 3.3. Association between gBRCA1/2 PV and Clinicopathological Characteristics

The clinicopathological features of participants with 19 g*BRCA1/2*-PV are shown in Table 1. Age at diagnosis, histological subtype, and disease stage were strong predictors of g*BRCA1/2* status. g*BRCA1/2*-PV was detected only in 5/66 (7.6%) of women with OC diagnosed before 55 years of age, but was found in 14/57 (24.6%) of those older than 55 years (*p* = 0.0121). No g*BRCA1/2*-PV was identified in women aged <40 years. The major histological subtype of OC in participants with g*BRCA1/2*-PV, especially g*BRCA1*-PV, was HGSC (13/19, 68.4%), although endometrioid and clear cell carcinomas were also observed in participants with g*BRCA1/2*-PV. Participants with g*BRCA1/2*-PV were more likely to be diagnosed at an advanced stage (FIGO stages III and IV) (*p* = 0.0002). Menopausal status did not differ between participants with g*BRCA1/2*-PV and those with wild-type g*BRCA1/2* (g*BRCA1/2*-WT). Individuals with OC and a personal history of BC tended to carry g*BRCA1/2*-PV, although the result was not significant owing to the small number of individuals available for study. Participants with g*BRCA1/2*-PV were more likely to have first- or second-degree relatives with BC (*p* = 0.0089) or all four HBOC-related cancers (*p* = 0.0379).

Of the 123 participants, 89 and 34 cases were initially treated with PDS and NAC, respectively (Supplementary Table S2, Supplementary Figure S1). In all participants, cases with g*BRCA1/2*-PV were more likely to be treated with NAC (*p* = 0.0123) because participants with OC with g*BRCA1/2*-PV were more likely to be diagnosed at an advanced stage. In participants with advanced-stage OC (FIGO III and IV), indeed, treatment did not differ according to g*BRCA1/2* status. Of the 38 participants with OC who were treated with doublet platinum-based chemotherapy (paclitaxel and carboplatin, TC) as NAC (*n* = 34) or chemotherapy because of suboptimal PDS (recurrence, *n* = 4) and had indicator lesions to be evaluated by imaging tests, favorable response (complete response, CR or partial response, PR) rates in participants with g*BRCA1/2*-PV and g*BRCA1/2*-WT were 100% (12/12 cases) and 88.5% (23/26 cases), respectively. A significantly higher recurrence rate was observed in participants with advanced-stage OC (*n* = 54) with g*BRCA1/2*-WT (19/35, 54.3%; *p* = 0.0232) than in those with g*BRCA1/2*-PV (4/19, 21.1%). Of the 23 participants with OC who experienced disease recurrence after initial therapy, 100% (4/4 cases) and 68.4% (13/19 cases) of participants with g*BRCA1/2*-PV and g*BRCA1/2*-WT, respectively, showed platinum-sensitive recurrence. In addition, 75% (3/4 cases) and 63.2% (12/19 cases) of participants with g*BRCA1/2*-PV and g*BRCA1/2*-WT, respectively, showed a favorable response to chemotherapy after OC recurrence. These observations demonstrate that participants with advanced-stage OC with g*BRCA1/2*-PV are likely to show a favorable response to chemotherapy, especially platinum-based chemotherapy.

**Figure 1:**
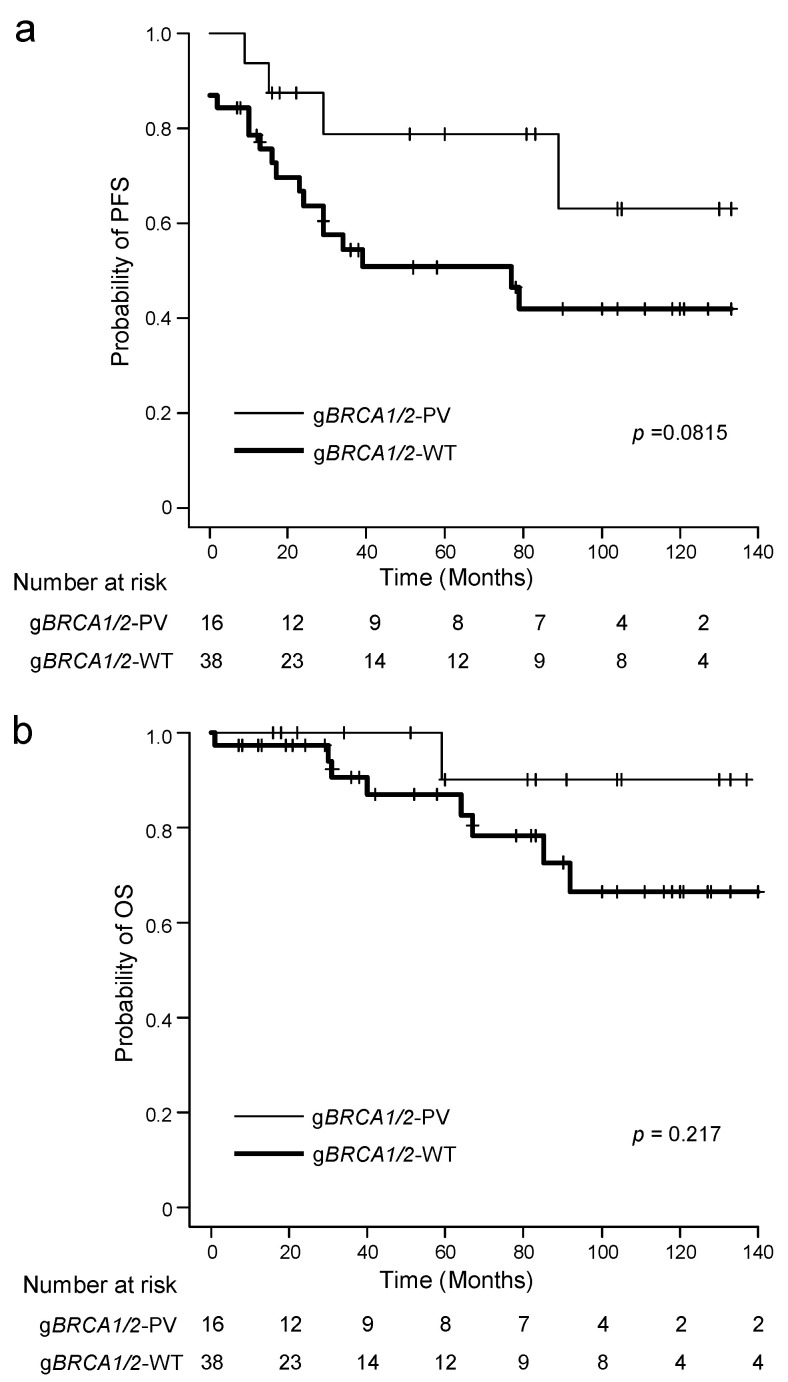
Survival outcomes of 54 participants with advanced-stage ovarian cancer (OC) with g*BRCA1/2* pathogenic variants (g*BRCA1/2*-PV) and wild type (g*BRCA1/2*-WT). (**a**) Progression-free survival (PFS) rate. (**b**) Overall survival (OS) rate.

Because most participants with g*BRCA1/2*-PV were diagnosed at an advanced stage (FIGO stage III or IV), we performed survival analyses on participants with advanced-stage OC (*n* = 54). Kaplan–Meier curves for PFS and OS according to g*BRCA1/2* status are shown in Figure 1. Participants with advanced-stage OC with g*BRCA1/2*-PV tended to have better PFS and OS than those with g*BRCA1/2*-WT, although the difference in PFS and OS probability was not significant. This difference in PFS probability was more remarkable in the NAC-treated advanced-stage OC group than in the PDS-treated advanced-stage OC group, although the difference was not significant as well (Supplementary Figure S2). In univariate analyses using a Cox proportional hazard regression model in participants with advanced-stage OC, none of the tested factors, including g*BRCA1/2* status, were significantly associated with PFS and OS, although g*BRCA1/2* -PV tended to be associated with improved PFS (hazard ratio = 0.418, 95% confidence interval = 0.121–1.113) and OS (hazard ratio = 0.289, 95% confidence interval = 0.016–1.579), particularly PFS (*p* values for PFS and OS are 0.084 and 0.173, respectively).

## 4. Discussion

This study reports on the status of g*BRCA1/2* in OC confined to a rural area of Japan, especially in a regional city with low migration and an aging population. The prevalence of g*BRCA1/2*-PV among 123 unbiased OC patients in this study (15.4%) is consistent with that reported previously in Japan (Supplementary Table S1) [6,7] and other countries [17,18]. The major histological subtype observed in OC patients with g*BRCA1/2*-PV was HGSC, which is also consistent with previous reports from Japan (Supplementary Table S1) [6,7]. The frequency of histological subtypes is known to be different between Japan and Western countries; the rate of clear cell carcinoma is higher in Japan (approximately 25%) than in the United States (approximately 6%), a four-fold difference [19,20]. The prevalence of g*BRCA1/2*-PV in HGSC and clear cell carcinoma in OC is also reported as higher and lower, respectively, in Japan than in Western countries [21,22]. Therefore, the total prevalence of g*BRCA1/2*-PV in all OC cases may be similar between Japan and Western countries despite the difference in the frequency of histologic subtypes. The prevalence of g*BRCA1/2*-PV in participants with HGSC in the current study was 27.7% (13/47), which is higher than that in studies in Caucasians [5,23] and is consistent with those in studies in the Japanese population [6,7]. Further studies are necessary to clarify why the prevalence of g*BRCA1/2*-PV in Japanese OC patients with HGSC is higher than that in Caucasians.

In contrast to previous studies from Japan, the prevalence of g*BRCA2*-PV was higher than that of g*BRCA1*-PV in unbiased OC patients in our study, although the total prevalence of g*BRCA1/2*-PV and the characteristics of cases with OC were similar among the studies [6,7]. The lifetime cumulative OC risk is known to be higher in individuals with g*BRCA1*-PV than in those with g*BRCA2*-PV [8], whereas the prevalence of pathogenic variants in a large Japanese female control population is four times higher in g*BRCA2* than in g*BRCA1* (0.17% vs. 0.04%, respectively) [24]. Therefore, the difference in the prevalence of each gene might be explained by genetic drift in the studied population or sampling bias due to the small number of participants. Among the 10 recurrently found g*BRCA1/2*-PV (in more than 10 families) in the Japanese Organization Of Hereditary Breast and Ovarian Cancer (JOHBOC) registry in Japan [16,25], only three variants, *BRCA1*:c.2800C>T, *BRCA2*:c.6952C>T, and *BRCA2*:c.9076C>T, were identified in our OC cases. In contrast, three g*BRCA2*-PV, two of which were observed in more than one case, found in our study had not been identified in previous studies of Japanese cases with OC [6,7] and BC [16,24]. In addition, among these three variants, NM_000059.4:c.3703C>T (p.Gln1235*) and NM_000059.4:c.7922dup (p.Phe2642Ilefs*3) have never been reported previously, suggesting that these are novel pathogenic variants and some g*BRCA2*-PV unique to the local area may exist in Tokushima prefecture located on the island of Shikoku, Japan.

An OCCR that was associated with a relative decrease and increase in the risks of BC and OC, respectively, had previously been determined in the *BRCA1* and *BRCA2* genes [15]. In the present study, g*BRCA1*-PV was more frequently observed within the OCCR (66.7%, 4/6 cases) compared with g*BRCA2*-PV (46.2%, 6/13 cases) (Table 2). Personal and family histories of BC were observed in OC patients with g*BRCA1/2*-PV, regardless of the variants within and outside the OCCR. In contrast, a family history of OC was observed only in OC patients with g*BRCA1/2*-PV within the OCCR, although only two OC patients with g*BRCA1/2*-PV, one in g*BRCA1* and the other in g*BRCA2*, had a family history of OC. No remarkable differences were observed in age, stage, or histological subtype according to variant location. These results are consistent with those previously reported in Japanese cases [7,26]. Two g*BRCA1/2*-PV, *BRCA1*:c.2800C>T and *BRCA2*:c.9076C>T (Table 2), which have been shown to have a relatively higher risk of OC among the 10 recurrently found g*BRCA1/2*-PV in the JOHBOC registry in Japan [25], were included in the list of g*BRCA1/2*-PV in the present study. Further analysis of the g*BRCA1/2*-PV location and its correlation with the risk of OC will be needed for accurate risk assessments of susceptible individuals and early detection of OC in g*BRCA1/2*-PV carriers.

Patients with OC with g*BRCA1/2*-PV have been reported to have both OS and PFS benefits comparable to those of patients with g*BRCA1/2*-WT [23,27,28,29,30,31,32], with g*BRCA2*-PV carriers having a better prognosis than g*BRCA1*-PV carriers [27]. In our study, improved PFS and OS, particularly PFS, were observed in advanced-stage OC patients with g*BRCA1/2*-PV compared with that in patients with g*BRCA1/2*-WT, although the differences were not significant between the two groups. Previously, g*BRCA1/2*-PV was shown to be associated with a better platinum response [23,33,34]. This higher platinum response in the g*BRCA1/2*-PV group was reported even for first-line chemotherapy [23,33], and this effect seems to persist in subsequent lines of chemotherapy. The present study demonstrated that (1) all advanced-stage OC patients with g*BRCA1/2*-PV showed a favorable response (CR or PR) after TC therapy in primary treatment (NAC or chemotherapy after suboptimal PDS, Supplementary Table S2), (2) the better PFS in advanced-stage OC patients with g*BRCA1/2*-PV compared to in those with g*BRCA1/2*-WT was more remarkable in primary NAC-treated patients using TC than in those treated with PDS (Supplementary Figure S2), and (3) a higher favorable response rate was observed in the g*BRCA1/2*-PV group, even in recurrent advanced-stage OC patients (Supplementary Table S2), although there were no significant differences between groups. Further prospective studies with larger sample sizes are needed to clarify the clinical characteristics, response to treatments, and outcomes in g*BRCA1/2*-PV-associated Japanese patients with OC.

This study has several limitations. First, the prevalence of g*BRCA1/2*-PV may have been underestimated in this series of patients because we included only variants with a definitely damaging impact on protein function according to the guidelines of the ACMG_AMP guidelines as a pathogenic variant [14]. However, the prevalence of g*BRCA1/2*-PV was similar to that reported in a previous study from Japan using the g*BRCA1/2* results tested by Myriad Genetics, Inc. [7]. Therefore, although some not-yet-characterized missense variants may prove damaging in g*BRCA1/2*, it is assumed that few g*BRCA1/2*-PV are missed. Second, the limited statistical power and selection bias due to the relatively small size of our cohort may have prevented the detection of a true association between g*BRCA1/2*-PV and clinicopathological features. Because Japanese national insurance coverage was extended to *BRCA1/2* genetic testing for all individuals with history of OC in 2020, the number of OC patients diagnosed with HBOC will increase rapidly in Japan, as it did in South Korea. A nationwide survey with a larger cohort is expected to clarify the association between gBRCA1/2-PV and clinicopathological features. Finally, this retrospective study did not include patients with poor prognoses who died very early.

## 5. Conclusions

We investigated the prevalence of g*BRCA1/2*-PV in unbiased OC patients and the clinical characteristics of g*BRCA1/2*-PV-associated OC patients in a rural area of Japan. Considering the results of the present study, genetic testing for g*BRCA1/2* status in all OC patients except mucinous and LGSC subtypes, which are g*BRCA1/2*-WT in most cases, may be useful not only for HBOC diagnosis and risk assessment of HBOC-related cancers in patients and their relatives, but also for estimating treatment response and outcome of OC patients. We also hope that our results will contribute to the implementation of genetic counseling before and/or after *BRCA1/2* genetic testing for optimal personalized medical management of patients with HBOC and their relatives.

## Figures and Tables

**Table 1:** Correlation between patient characteristics and germline variant status of *BRCA1/2* in 123 patients with ovarian cancer (OC).

Characteristic	*n*	g*BRCA1/2* Variant Status				*p* Value * (g*BRCA1/2*-PV vs. WT)
		g*BRCA1*-PV	g*BRCA2*-PV	g*BRCA1/2*-PV	g*BRCA1/2*-WT	
**Total**	123	6 (4.9%)	13 (10.5%)	19 (15.4%)	104 (84.6%)	
**Age at diagnosis, years**						<55 vs. ≥55
Median (range)	54 (28–87)	56.5 (44–71)	60 (46–67)	60 (44–71)	53 (28–85)	**0.0224**
<40	15 (12.2%)	0 (0%)	0 (0%)	0 (0%)	15 (14.4%)	
≥40, <55	47 (38.2%)	3 (50.0%)	2 (15.4%)	5 (26.3%)	42 (40.4%)	
≥55	61 (49.6%)	3 (50.0%)	11 (84.6%)	14 (73.7%)	47 (45.2%)	
**Menopausal status**						Pre vs. Post
Pre-menopausal	46 (37.4%)	2 (33.3%)	2 (15.4%)	4 (21.1%)	42 (40.4%)	0.1856
Post-menopausal	71 (57.7%)	3 (50.0%)	10 (76.9%)	13 (68.4%)	58 (55.8%)	
Unknown	6 (4.9%)	1 (16.7%)	1 (7.7%)	2 (10.5%)	4 (3.8%)	
**Histological type**						HGSC vs. non-HGSC
HGSC	47 (38.2%)	5 (83.3%)	8 (61.5%)	13 (68.4%)	34 (32.7%)	**0.0046**
LGSC	5 (4.1%)	0 (0%)	0 (0%)	0 (0%)	5 (4.8%)	
Endometrioid	18 (14.6%)	0 (0%)	3 (23.1%)	3 (15.8%)	15 (14.4%)	
Clear cell	28 (22.8%)	0 (0%)	1 (7.7%)	1 (5.3%)	27 (26.0%)	
Mucinous	13 (10.6%)	0 (0%)	0 (0%)	0 (0%)	13 (12.5%)	
Others	12 (9.8%)	1 (16.7%)	1 (7.7%)	2 (10.5%)	10 (9.6%)	
**FIGO stage**						Stage I + II vs. III + IV
I	53 (43.1%)	0 (0%)	2 (15.4%)	2 (10.5%)	51 (49.0%)	**0.0002**
II	16 (13.0%)	0 (0%)	1 (7.7%)	1 (5.3%)	15 (14.4%)	
III	50 (40.7%)	6 (100%)	9 (69.2%)	15 (78.9%)	35 (33.7%)	
IV	4 (3.3%)	0 (0%)	1 (7.7%)	1 (5.3%)	3 (2.9%)	
**Personal history of breast cancer**						Presence vs. Absence
Presence	11 (8.9%)	1 (16.7%)	2 (15.4%)	3 (15.8%)	8 (7.7%)	0.3733
Absence	112 (91.1%)	5 (83.3%)	11 (8.5%)	16 (84.2%)	96 (92.3%)	
**Family history** **(including first- and second-degree relatives)** **of HBOC-associated cancers**						Presence vs. Absence
Breast cancer	23 (18.7%)	3 (50.0%)	5 (38.5%)	8 (42.1%)	15 (14.4%)	**0.0089**
Ovarian cancer	5 (4.1%)	1 (16.7%)	1 (7.7%)	2 (10.5%)	3 (2.9%)	0.1701
Prostate cancer	16 (13.0%)	1 (16.7%)	3 (23.1%)	4 (21.1%)	12 (11.5%)	0.2708
Pancreatic cancer	10 (8.1%)	0 (0%)	1 (7.7%)	1 (5.3%)	9 (8.7%)	1.0000
All HBOC-associated cancers	44 (15.8%)	4 (66.7%)	7 (53.8%)	11 (57.9%)	33 (31.7%)	**0.0379**

PV, pathogenic variant; WT, wild-type; HGSC, high-grade serous; LGSC, low-grade serous; FIGO, International Federation of Gynecology and Obstetrics; HBOC, hereditary breast and ovarian cancer. * **Bold font** indicates significant values (*p*  <  0.05) obtained by analysis using Pearson’s χ^2^-test or Fisher’s exact test.

**Table 2:** List of g*BRCA1/2* pathogenic variants and clinicopathological features of patients with ovarian cancer (OC) with these variants.

No.	Age	Stage	Histological Subtype	Variant Position (OCCR)	Nucleotide Change	Amino Acid Change	Hirasawa et al. [6] (OC)	CHARLOTTE Study [7] (OC)	Yoshimura et al. [16] (BC)	Personal History of BC	Family History of Cancers			
											BC	OC	PanC	PrC
***BRCA1* (NM_007294.4)**														
1	61	III	HGSC	within	**c.2800C>** **T**	p.Gln934*	3 cases	4 cases	28 cases	+	−	−	−	−
2	71	III	HGSC	within	c.3505_3509del	p.Asp1169*	0 case	0 case	2 cases	−	−	−	−	−
3	49	III	HGSC	within	c.3505_3509del	p.Asp1169*	0 case	0 case	2 cases	−	−	+	−	−
4	44	III	HGSC	within	c.3505_3509del	p.Asp1169*	0 case	0 case	2 cases	−	+	−	−	+
5	67	III	HGSC	outside	c.5161C>T	p.Gln1721*	0 case	0 case	3 cases	−	+	−	−	−
6	52	III	Others	outside	c.5161C>T	p.Gln1721*	0 case	0 case	3 cases	−	+	−	−	−
***BRCA2* (NM_000059.3)**														
7	56	III	Endometrioid	outside	c.805dup	p.Thr269Asnfs*7	0 case	0 case	1 case	+	−	−	−	+
8	46	III	HGSC	outside	c.2830A>T	p.Lys944*	0 case	1 case	0 case	−	−	−	−	−
9	62	I	HGSC	within	c.3703C>T	p.Gln1235*	0 case	0 case	0 case	−	−	−	−	−
10	60	IV	Clear cell	within	c.5645C>A	p.Ser1882*	0 case	0 case	7 cases	−	−	−	−	−
11	72	III	HGSC	within	c.5645C>A	p.Ser1882*	0 case	0 case	7 cases	−	+	−	−	−
12	46	III	Endometrioid	within	c.6649A>T	p.Lys2217*	0 case	0 case	1 case	+	+	+	−	−
13	66	III	HGSC	within	**c.6952C>T**	p.Arg2318*	1 case	11 cases	18 cases	−	+	−	−	−
14	60	III	HGSC	within	**c.6952C>T**	p.Arg2318*	1 case	11 cases	18 cases	−	−	−	−	−
15	60	I	Clear cell	outside	c.7758G>A	p.Trp2586*	0 case	0 case	0 case	−	+	−	+	−
16	59	II	Endometrioid	outside	c.7758G>A	p.Trp2586*	0 case	0 case	0 case	−	−	−	−	−
17	67	III	HGSC	outside	c.7922dup	p.Phe2642Ilefs*3	0 case	0 case	0 case	−	−	−	−	−
18	67	III	HGSC	outside	c.7922dup	p.Phe2642Ilefs*3	0 case	0 case	0 case	−	+	−	−	+
19	59	III	HGSC	outside	**c.9076C>T**	p.Gln3026*	0 case	1 case	10 cases	−	−	−	−	+

OCCR, ovarian cancer cluster region; BC, breast cancer; OC, ovarian cancer; PanC, pancreatic cancer; PrC, prostate cancer; HGSC, High-grade serous. **Bold font** indicates that the pathogenic variant is recurrently found (more than 10 families) in the Japanese Organization of Hereditary Breast and Ovarian Cancer (JOHBOC) registry in Japan.

## Data Availability

The datasets of germline pathogenic variants in BRCA1/2 generated during and/or analyzed during the current study are available in the ClinVar repository, https://www.ncbi.nlm.nih.gov/clinvar/submitters/508150/ (accessed on 12 May 2022). All other datasets including clinicopathological data of participants generated during and/or analyzed during the current study are available from the corresponding author on reasonable request.

## Supplementary Materials

The following supporting information can be downloaded at: https://www.mdpi.com/article/10.3390/genes13061085/s1, Table S1: Demographic and clinical characteristics in patients with ovarian cancer (OC) included in the current study and two studies reported from Japan; Table S2: Clinical course and response to chemotherapy by g*BRCA1/2* variant status; Figure S1: Therapies and responses to treatment for 123 patients with ovarian cancer (OC); Figure S2: Progression-free survival (PFS) rates in patients with advanced-stage ovarian cancer (OC) according to primary treatments. (a) primary debulking surgery (PDS), (b) neoadjuvant chemotherapy (NAC) followed by interval debulking surgery (IDS).
