# Supplementary material for: Prevalence of Pathogenic Germline BRCA1/2 Variants and Their Association with Clinical Characteristics in Patients with Epithelial Ovarian Cancer in a Rural Area of Japan
Source: Genes (Basel). 2022 Jun 18;13(6):1085. doi: 10.3390/genes13061085 (PMC9223158; doi:10.3390/genes13061085)
Supplement: Supplementary file 1 [file genes-13-01085-s001.zip › genes-1747299-supplementary.pdf]

genes

MDPI

Supplementary Materials

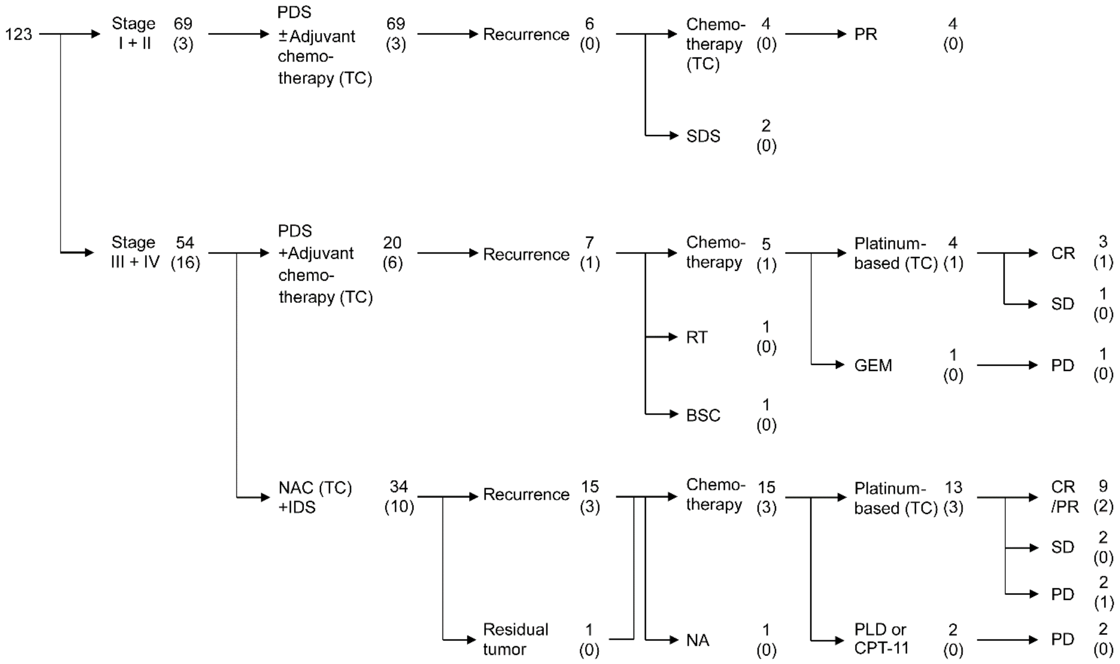

Figure S1: Therapies and responses to the treatments for 123 OC patients

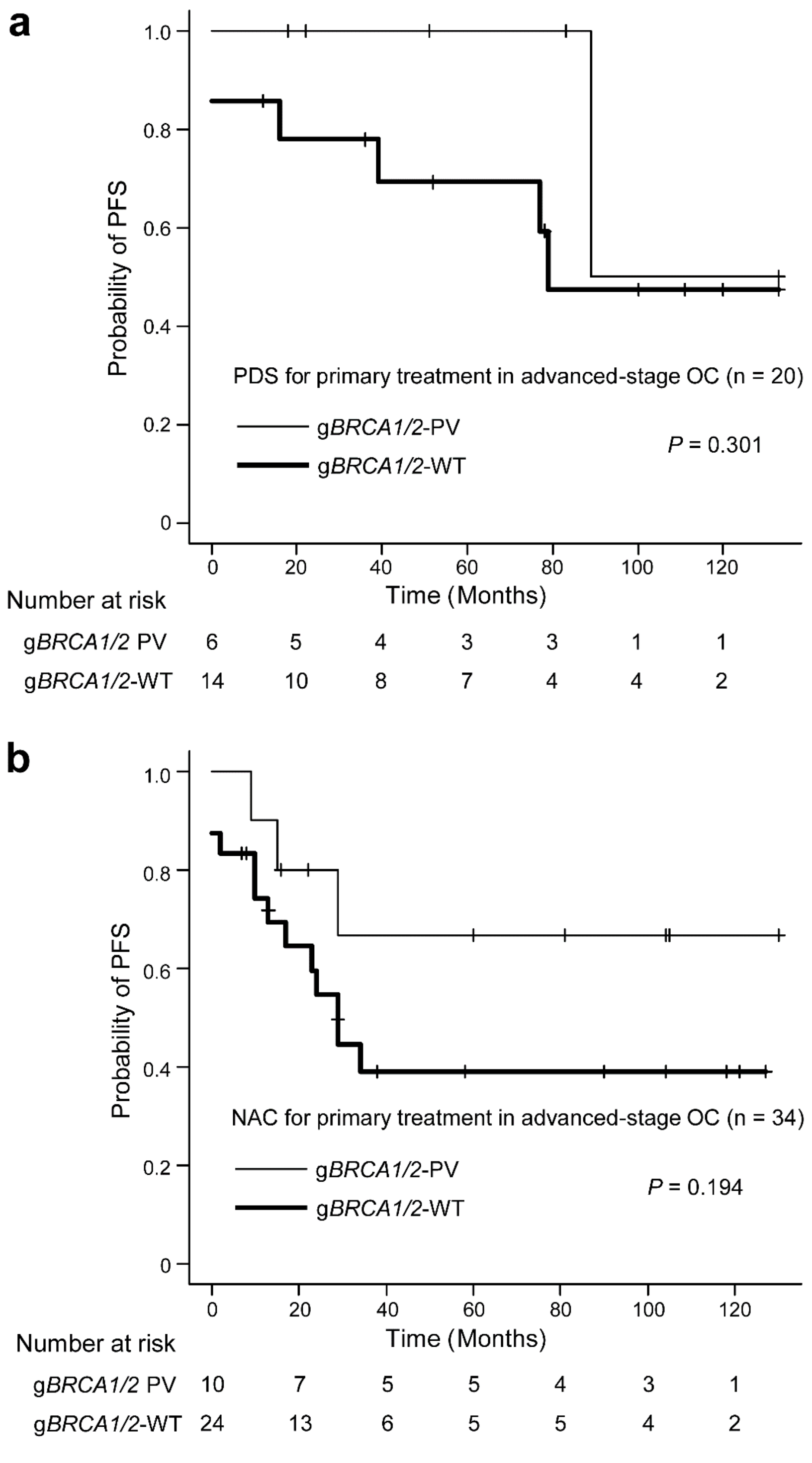

Figure S2: Progression-free survival (PFS) rates in advanced-stage OC patients according to primary treatments. (a) PDS, (b) NAC-IDS

Supplementary Table S1 Demographic characteristics and pathogenic BRCA variants of ovarian cancer patients in a combination of three studies in Japan

Characteristics	Present study	Hirasawa et al. [Ref. 6]	Charlotte study [Ref. 7]	Total
**Total (*n*)**	123	230	634	987
**Age, median, range (years)**	55 (28–85)	54 (27–87)	56.9 (mean) (24–89)	NA
<40	0/15 (0.0%)	4/18 (22.2%)	4/41 (9.8%)	8/74 (11.0%)
≥40, <55	5/47 (10.6%)	15/99 (15.2)	89/593 (15.0%)	20/156 (13.2%)
≥55	14/61 (30.0%)	8/113 (7.1%)		22/174 (12.9%)
**Menopausal status**				
Pre-menopausal	4/46 (8.7%)	NA	70/169 (41.4%)	74/215 (34.3%)
Post-menopausal	13/71 (18.3%)	NA	22/462 (4.8%)	35/533 (6.6%)
Unknown	2/6 (33.3%)	NA	1/3 (33.3%)	3/9 (33.3%)
**Histological type**				
HGSC	13/47 (27.7%)	22/74 (29.7%)	78/274 (28.5%)	113/395 (28.6%)
LGSC	0/5 (0.0%)	0/3 (0.0%)	1/5 (20.0%)	1/13 (7.7%)
Endometrioid carcinoma	3/18 (16.7%)	1/58 (1.7%)	8/120 (6.7%)	12/196 (6.1%)
Clear cell carcinoma	1/28 (3.6%)	2/71 (2.8%)	4/187 (2.1%)	7/286 (2.4%)
Mucinous carcinoma	0/13 (0.0%)	0/18 (0.0%)	0/19 (0.0%)	0/50 (0%)
Others	2/12 (16.7%)	1/6 (16.7%)	2/29 (6.9%)	5/47 (10.6%)
**Stage**				
I	2/53 (3.8%)	2/107 (1.9%)	8/236 (3.4%)	12/396 (3%)
II	1/16 (6.3%)	4/25 (16.0%)	7/71 (9.9%)	12/112 (10.7%)
III	15/50 (30.0%)	14/80 (17.5%)	62/244 (25.4%)	91/374 (24.3%)
IV	1/4 (25.0%)	7/18 (38.9%)	16/80 (20.0%)	24/102 (23.5%)
Other	—	—	0/3 (0.0%)	
**Personal history of breast cancer**				
Presence	3/11 (27.3%)	2/6 (33.3%)	14/30 (46.7%)	42 (30.2%)
**Familial history (including first- and second-degree relatives) of HBOC-associated cancers**				
Breast cancer	8/23 (34.8%)	5/22 (22.7%)	37/118 (31.4%)	50/163 (30.7%)
Pancreatic cancer	1/10 (10.0%)	NA	12/53 (22.6%)	13/112 (11.6%)
Ovarian cancer	2/5 (40.0%)	5/22 (22.7%)	23/36 (63.9%)	30/134 (22.4%)
Prostate cancer	4/16 (40.0%)	NA	6/32 (18.8%)	30/134 (22.4%)
**Germline pathogenic variants**				
BRCA1	6 (4.9%)	19 (8.3%)	63 (9.9%)	88 (8.9%)
BRCA2	13 (10.5%)	8 (3.5%)	30 (4.7%)	51 (5.2%)
BRCA1 or *BRCA2*	19 (15.4%)	27 (11.7%)	93 (14.7%)	139 (14.1%)

Values are n (%), unless otherwise stated.
NA, not available; HGSC, high-grade serous carcinoma; LGSC, low-grade serous carcinoma; FIGO, International Federation of Gynecology and Obstetrics; HBOC, hereditary breast and ovarian cancer syndrome.

Supplementary Table S2 The clinical course and clinical response to chemotherapy

	n	g*BRCA1*/2 variant status		*P*-value*
		PV	WT	
**Primary treatment strategy in all cases**	123	19	104	PDS *vs*. NAC
Primary debulking surgery (PDS)	89	9 (47.4%)	80 (76.9%)	**0.0123**
Neoadjuvant chemotherapy (NAC)	34	10 (52.6%)	24 (23.1%)	
**Primary treatment in advanced-stage cases (stage III and IV)**	54	16	38	PDS *vs*. NAC
PDS	20	6 (37.5%)	14 (36.8%)	1.000
NAC	34	10 (62.5%)	24 (63.2%)	
**Clinical response to TC therapy in primary treatment**	38 (34 NAC + 4 suboptimal PDS)	12	26	PR/CR *vs*. SD/PD
PR+CR	35	12 (100%)	23 (88.5%)	0.5377
SD	2	0 (0%)	2 (7.7%)	
PD	1	0 (0%)	1 (3.8%)	
**Recurrence in advanced-stage cases**	54	19	35	No *vs*. YES
No	31	15 (78.9%)	16 (45.7%)	**0.0232**
Yes	23	4 (21.1%)	19 (54.3%)	
Platinum-sensitive recurrence	17 (73.9%)	4 (100%)	13 (68.4%)	0.5392
Platinum-resistant recurrence	6 (26.1%)	0 (0%)	6 (31.6%)	
**Clinical response to chemotherapy in recurrent advanced-stage cases**	20	4	16	PR/CR *vs*. SD/PD
PR+CR	12	3 (75%)	9 (56.2%)	0.6186
SD	3	0 (0%)	3 (18.8%)	
PD	5	1 (25%)	4 (25.0%)	

PV, pathogenic variants; WT, wild-type; TC, paclitaxel-carboplatin; PR, partial response; CR, complete response; SD, stable disease; PD, progressive disease.
***Bold font** indicates a statistically significant value ($P < 0.05$) obtained by analysis with Pearson's χ2-test or Fisher's exact test.
